# Topology testing of phylogenies using least squares methods

**DOI:** 10.1186/1471-2148-6-105

**Published:** 2006-12-06

**Authors:** Aleksandra Czarna, Rafael Sanjuán, Fernando González-Candelas, Borys Wróbel

**Affiliations:** 1 Department of Marine Genetics and Biotechnology, Institute of Oceanology, Polish Academy of Sciences, Powstanców Warszawy 55, PL-81712 Sopot, Poland; 2 Instituto de Biología Molecular y Celular de Plantas, CSIC/Universidad Politécnica de Valencia, Valencia, Spain; 3 Institut Cavanilles de Biodiversitat i Biologia Evolutiva, Universitat de València, Spain

## Abstract

**Background:**

The least squares (LS) method for constructing confidence sets of trees is closely related to LS tree building methods, in which the goodness of fit of the distances measured on the tree (patristic distances) to the observed distances between taxa is the criterion used for selecting the best topology. The generalized LS (GLS) method for topology testing is often frustrated by the computational difficulties in calculating the covariance matrix and its inverse, which in practice requires approximations. The weighted LS (WLS) allows for a more efficient albeit approximate calculation of the test statistic by ignoring the covariances between the distances.

**Results:**

The goal of this paper is to assess the applicability of the LS approach for constructing confidence sets of trees. We show that the approximations inherent to the WLS method did not affect negatively the accuracy and reliability of the test both in the analysis of biological sequences and DNA-DNA hybridization data (for which character-based testing methods cannot be used). On the other hand, we report several problems for the GLS method, at  least for the available implementation. For many data sets of biological sequences, the GLS statistic could not be calculated. For some data sets for which it could, the GLS method included all the possible trees in the confidence set despite a strong phylogenetic signal in the data. Finally, contrary to WLS, for simulated sequences GLS showed undercoverage (frequent non-inclusion of the true tree in the confidence set).

**Conclusion:**

The WLS method provides a computationally efficient approximation to the GLS useful especially in exploratory analyses of confidence sets of trees, when assessing the phylogenetic signal in the data, and when other methods are not available.

## Background

From a statistical point of view, the inference of phylogenies is similar to the estimation of an unknown quantity in the presence of uncertainty. Given the intrinsic uncertainty in solving phylogenetic relationships from limited (in size and numbers) samples, it is necessary to assume that phylogenetic estimates are subject to stochastic and systematic errors [1]. Consequently, the correct answer to a phylogenetic problem is not a single estimate – one topology optimal under the assumptions of a particular phylogenetic reconstruction method. Rather, it is more appropriate to derive a set of phylogenies that capture the uncertainty about the solution to the phylogenetic reconstruction problem from the available data.

Several statistical procedures have been proposed to test trees and to construct confidence sets of topologies derived from sequence data. These procedures include the bootstrap selection probability (BP) of Felsenstein [2], and its modification [3], the Approximate Unbiased (AU) test, which reduces test bias and improves the accuracy and the simplicity of implementation. However, many concerns have been raised about the use of BP [4-6]. Statistical tests of phylogenies based on maximum likelihood (which also involve bootstrapping, either parametric or non-parametric) include the Kishino-Hasegawa (KH; [7]) test, which was later modified by Shimodaira and Hasegawa (SH; [8]) to take into account test multiplicity, and the Swofford-Olsen-Wadden-Hillis (SOWH; [9,10]) test. Unfortunately, in some situations these tests give contradictory results [10,11]: the SH test seems to be too conservative, especially in comparison to the SOWH test, which often rejects all but the maximum likelihood topology. Strimmer and Rambaut [11] have argued that these discrepancies may be caused by model misspecification; however, the solution they proposed, the expected likelihood weights (ELW) test, shares with the SOWH the inconvenience of being computationally intensive.

The generalized least squares (GLS) method for constructing confidence sets [12,13] is closely related to least squares (LS) tree building methods [14,15], in which the goodness of fit of the distances measured on the tree (patristic distances) to the observed distances between taxa is the criterion used for selecting the best topology. These methods do not require expensive calculations, which make them applicable to the analysis of very large data sets. However, the use of the GLS test for such data sets is often frustrated by the computational difficulties in calculating the covariance matrix and its inverse.

The GLS test [13] is based on the assumption that the evolutionary (observed) distance between each particular pair of taxa takes a value drawn from a normal distribution centered on the patristic distance. Under a null hypothesis that a given topology is true, the GLS test statistic:

∑i,j,k,lwij,kl(dij−eij)(dkl−ekl)     (1)
 MathType@MTEF@5@5@+=feaafiart1ev1aaatCvAUfKttLearuWrP9MDH5MBPbIqV92AaeXatLxBI9gBaebbnrfifHhDYfgasaacH8akY=wiFfYdH8Gipec8Eeeu0xXdbba9frFj0=OqFfea0dXdd9vqai=hGuQ8kuc9pgc9s8qqaq=dirpe0xb9q8qiLsFr0=vr0=vr0dc8meaabaqaciaacaGaaeqabaqabeGadaaakeaadaaeqbqaaiabdEha3naaBaaaleaacqWGPbqAcqWGQbGAcqGGSaalcqWGRbWAcqWGSbaBaeqaaaqaaiabdMgaPjabcYcaSiabdQgaQjabcYcaSiabdUgaRjabcYcaSiabdYgaSbqab0GaeyyeIuoakmaabmaabaGaemizaq2aaSbaaSqaaiabdMgaPjabdQgaQbqabaGccqGHsislcqWGLbqzdaWgaaWcbaGaemyAaKMaemOAaOgabeaaaOGaayjkaiaawMcaamaabmaabaGaemizaq2aaSbaaSqaaiabdUgaRjabdYgaSbqabaGccqGHsislcqWGLbqzdaWgaaWcbaGaem4AaSMaemiBaWgabeaaaOGaayjkaiaawMcaaiaaxMaacaWLjaWaaeWaaeaacqaIXaqmaiaawIcacaGLPaaaaaa@589A@

follows a chi-square distribution, provided the distances are (approximately) normal (for example, they are maximum likelihood evolutionary distances; [13]). In this formulation, *d*_*ij *_are the evolutionary distances, *e_ij_*are patristic distances (distances measured on the tree between taxa *i *and *j*), and *w*_*ij, kl *_are entries in the inverted matrix of variances and covariances of the distances.

The problem of estimating the covariance matrix has been only recently solved by Susko [13]. Two methods for estimation of the variances and covariances were proposed: the sample average method and bootstrap estimation. Only the former was implemented; still, the analysis of biological sequences showed that both give very close estimates [13]. Both methods require access to sequence data. However, the advantage of distance methods of phylogenetic reconstruction, including those using the LS approach, over character-based methods is that the distances need not be derived from sequences, and even if they are, access to the character data is not necessary. In principle, LS methods could be used for such data as an alternative to bootstrapping [16] and jackknifing methods [17].

The calculation of the GLS statistic requires inverting the covariance matrix, which is not always possible. A solution suggested by Susko [18] is to ignore the entries in the matrix that are close to zero, which results in a more conservative test. If the covariances are completely ignored, and only the values in the diagonal (the variances) are used, the sum gives a weighted least squares (WLS) statistic:

∑i,jwij(dij−eij)2     (2)
 MathType@MTEF@5@5@+=feaafiart1ev1aaatCvAUfKttLearuWrP9MDH5MBPbIqV92AaeXatLxBI9gBaebbnrfifHhDYfgasaacH8akY=wiFfYdH8Gipec8Eeeu0xXdbba9frFj0=OqFfea0dXdd9vqai=hGuQ8kuc9pgc9s8qqaq=dirpe0xb9q8qiLsFr0=vr0=vr0dc8meaabaqaciaacaGaaeqabaqabeGadaaakeaadaaeqbqaaiabdEha3naaBaaaleaacqWGPbqAcqWGQbGAaeqaaaqaaiabdMgaPjabcYcaSiabdQgaQbqab0GaeyyeIuoakmaabmaabaGaemizaq2aaSbaaSqaaiabdMgaPjabdQgaQbqabaGccqGHsislcqWGLbqzdaWgaaWcbaGaemyAaKMaemOAaOgabeaaaOGaayjkaiaawMcaamaaCaaaleqabaGaeGOmaidaaOGaaCzcaiaaxMaadaqadaqaaiabikdaYaGaayjkaiaawMcaaaaa@469F@

In this formulation, the distances are treated as independent to avoid computational difficulties. Again, one can view this simplification as avoiding the division by numbers very close to zero, which results in a test statistic smaller than the corresponding GLS statistic, and consequently fewer rejections (a more conservative test).

Although the phylogenetic distances are *a priori *not independent because taxa share evolutionary history, we have shown in a previous work that ignoring the covariances does not have drastic consequences for the reliability or the accuracy of the LS approach to interior branch testing in phylogenetic trees derived from sequences [6].

In this work we investigate the applicability of LS methods for construction of confidence sets for topologies. We start by re-analyzing a well-known data set of six long sequences of mammalian mitochondrial proteins, for which the GLS approach has been previously used. We then explore the size of the confidence sets obtained with the GLS and WLS methods using a database of nucleotide sequences. Each data set in the database consisted of eight sequences chosen to minimize the effects of model misspecification. This was necessary since the existing implementation of the GLS method allows its application only when simple models of nucleotide substitutions are used (the implementation of the WLS method we present does not have such limitations). We also present a simulation analysis in order to investigate both the size of confidence sets and the coverage of the LS methods. Finally, we apply the WLS method to two data sets for which the GLS method could not be used: (i) a large number of short viral sequences in which testing alternative phylogenies is key in including or excluding patients from a nosocomial outbreak of hepatitis C, and (ii) DNA/DNA hybridization data, where neither the GLS method nor other methods of topology testing which require access to character data can be used.

The goal of this paper is to assess the applicability of the LS approach to construct confidence sets of trees from biological data. We will explore the consequences in terms of accuracy and reliability of the approximations inherent to both the GLS and WLS method.

## Results

### Mammalian mitochondrial protein sequences

We will first consider the mammalian mitochondrial protein data set originally analyzed by Shimodaira and Hasegawa [8], and then by Goldman, Anderson and Rodrigo [10], Shimodaira [3], Strimmer and Rambaut [11], and Susko [13]. This data set consists of 3414 aligned amino acids from six mammalian species: cow, harbor seal, human, mouse, opossum, and rabbit. We have used the generalized least squares test proposed by Susko [13] implemented in the GLSPROT program [13], which uses the PAM substitution model [19]. Table 1 shows the comparison of the results obtained with GLS and WLS tests, and three tests based on likelihood (SH, KH and ELW tests), assuming the PAM substitution model. All possible 105 unrooted topologies for six species were considered.

**Table 1:** Confidence sets of trees derived from mammalian mitochondrial protein data

**Tree**	**ELW**	**KH**	**SH**	**GLS**	**WLS**
(opposum,(mouse,(rabbit,((seal, cow), human))))	**0.2683**	**0.3290**	**0.9310**	**0.4097**	**0.6825**
(opposum,(mouse,((rabbit,(seal, cow)), human)))	**0.5128**	**1.0000**	**1.0000**	**0.3800**	**0.7081**
(opposum,(mouse,((seal, cow),(rabbit, human))))	**0.0624**	**0.1580**	**0.8610**	**0.3533**	**0.6788**
(opposum,((mouse,(rabbit,(seal, cow))), human))	**0.0569**	**0.1010**	**0.8010**	**0.0502**	**0.1490**
(opposum,((rabbit,(seal, cow)),(mouse, human)))	0.0012	0.0090	**0.5960**	**0.0502**	**0.1491**
(opposum,(rabbit,(mouse,((seal, cow), human))))	0.0000	0.0050	**0.2910**	0.0244	**0.0673**
(opposum,((rabbit, mouse),((seal, cow), human)))	0.0039	**0.0520**	**0.4980**	0.0244	**0.0672**
(opposum,((seal, cow),((rabbit, mouse), human)))	0.0037	0.0410	**0.4570**	0.0135	0.0494
(opposum,(((seal, cow),(rabbit, mouse)), human))	**0.0877**	**0.1460**	**0.6910**	0.0135	0.0496
(opposum,(rabbit,((seal, cow),(mouse, human))))	0.0000	0.0000	**0.2230**	0.0130	0.0496
(opposum,((seal, cow),(mouse, (rabbit, human))))	0.0004	0.0150	**0.3390**	0.0130	0.0494
(opposum,((seal, cow),(rabbit,(mouse, human))))	0.0026	0.0180	**0.3800**	0.0130	0.0495
(opposum,((mouse,(seal, cow)),(rabbit, human)))	0.0000	0.0000	**0.2000**	0.0130	0.0493
(opposum,(rabbit,((mouse,(seal, cow)), human)))	0.0000	0.0000	**0.1380**	0.0130	0.0494
(opposum,((rabbit,(mouse,(seal, cow))), human))	0.0000	0.0120	**0.3890**	0.0130	0.0495
(opposum,(mouse,(rabbit,(seal,(cow, human)))))	0.0000	0.0010	0.0480	0.0000	0.0000
(opposum,(mouse,(rabbit,(cow,(seal, human)))))	0.0000	0.0030	0.0480	0.0000	0.0000
(opposum,((rabbit, mouse),(seal,(cow, human))))	0.0000	0.0010	0.0010	0.0000	0.0000
(opposum,(rabbit,(mouse,(seal,(cow, human)))))	0.0000	0.0000	0.0010	0.0000	0.0000
(opposum,(rabbit,(mouse,(cow,(seal, human)))))	0.0000	0.0000	0.0010	0.0000	0.0000

Aminoacid sequences were taken from six mammalian species: *Homo sapiens *(human), *Phoca vitulina *(seal), *Bos taurus *(cow) *Oryctolagus cuniculus *(rabbit), *Mus musculus *(mouse), and *Didelphis virginiana *(opposum). The tree believed to be the best estimate of mammalian phylogeny [40] has been underlined. Values in bold indicate the trees included in the 0.95 confidence set: the trees with the highest confidence levels which add up to 0.95 for the expected likelihood weights (ELW) test, and the trees with *P*-values above 0.05 for one-tailed Kishino-Hasegawa (KH) test, Shimodaira-Hasegawa (SH) test, generalised least squares (GLS) test, and weighted least squares test (WLS).

The GLS 0.95 confidence set, as has been previously shown by Susko [13] for this data set, is formed by just five trees. As expected, the WLS gives more conservative results, and includes two more topologies in the 0.95 set. Both the GLS and WLS 0.99 confidence sets include all the trees containing the (seal, cow) cluster. The same 15 trees form the 0.95 confidence set of the SH test, which for this data set is the most conservative. The sets of topologies accepted by ELW and KH tests at the 0.95 level include five and six trees, respectively.

With a more appropriate mtREV+G substitution model [20] the SH, KH and ELW methods gave similar results (for the ELW, tree number four was excluded, for the KH, tree number five was included, and number seven excluded from the 0.95 confidence set). The WLS method put all 15 trees with the (sea, cow) cluster in the 0.95 confidence set. It would be interesting to know how the GLS results would be affected by using a different substitution model, but as was mentioned above, only the PAM model has been implemented in the existing software [13].

### The size of LS confidence sets for nucleotide sequence data

The high computational efficiency of LS methods allows investigating the size of the confidence sets. For a small number of taxa, *P*-values can be obtained for all possible topologies. This becomes infeasible when the number of trees increases, but approximate confidence sets can be obtained by focusing on the trees with *P*-values above some threshold during the heuristic search. In order to evaluate and compare the sizes of the confidence sets obtained with the GLS method and our computationally simpler approach, we have constructed a database of eight-species data sets of nucleotide sequences obtained from EMBL-ALIGN [21]. Gaps and positions of doubtful homology in the multiple alignments were removed using Gblocks [22]; only alignments longer than 1000 nucleotides were kept: 108 out of 539 in the EMBL-ALIGN database.

Finding the data sets for which the GLS statistic could be calculated proved to be a very difficult task. We iteratively constructed eight-taxon subsets of each data set in the EMBL-ALIGN database until we found a subset for which the GLS statistic could be calculated. For many data sets, such a subset could be found only after several thousands of subsets were considered. Even so, it was necessary to use the GLSDNA_EIG routine, which approximates the GLS calculations when the covariance matrix has small eigenvalues [18].

The Felsenstein84 [23] substitution model is the most complex model in the available implementation of the GLS method [13]. To avoid the problem of using an inappropriate substitution model, we considered only the data sets for which the Akaike Information Criterion difference from the model with the minimum AIC was less than 10 for the F84 model. The AIC difference was calculated using ModelTest [24].

Table 2 shows the results for 16 data sets for which the eigenvalue cutoff in GLS calculations was less than 10^-10^. When the LS statistic was calculated using the WLS approximation, the size of the confidence sets was always smaller than the SH confidence set and, surprisingly, often smaller than the GLS set. For one data set (7, obtained from ALIGN_000623), the GLS test did not reject any topology, while the WLS confidence set consisted of 33 trees. It is unlikely that this was caused by the use of an inappropriate nucleotide substitution model: indeed, the F84 model was judged optimal for this data set according to the Akaike Information Criterion. When both LS methods included all the possible topologies in the confidence set, other pieces of evidence supported a low phylogenetic signal in the data: Table 2 lists the percentage of four-taxon subsets for which the star topology was the ML solution calculated using TREE-PUZZLE [25].

**Table 2:** Confidence sets of trees derived from eight-taxon data sets obtained from the EMBL-ALIGN database.

**Data set number**	**EMBL-ALIGN accession number**	**Sequences included**	**length**	**ΔAIC for F84**	**% of unresolved quartets**	**Number of trees in the 0.95 confidence set**			
						**SH**	**ELW**	**GLS**	**WLS**

1	ALIGN_000002	1,2,3,4,5,6,7,8	1632	6.4141	22.9	141	14	9	135
2	ALIGN_000205	2,3,4,6,8,10,11,12	1386	6.2104	4.3	15	6	18	9
3	ALIGN_000297	2,3,4,6,15,16,17,19	1167	0.0000	31.4	315	258	315	315
4	ALIGN_000397	2,3,4,6,7,8,9,10	1662	0.0000	24.3	2745	328	10395	10206
5	ALIGN_000398	1,2,3,4,5,6,7,8	1656	3.1567	0.0	477	20	815	77
6	ALIGN_000521	1,2,3,4,5,6,7,8	1325	8.4751	5.7	135	11	105	107
7	ALIGN_000623	2,3,4,5,6,10,11,12	1312	0.0000	0.0	380	20	10395	33
8	ALIGN_000628	2,3,4,5,7,13,17,31	1385	0.3071	0.0	141	5	117	21
9	ALIGN_000767	2,3,4,5,6,7,8,10	1386	6.6421	4.3	15	6	9	9
10	ALIGN_000771	1,2,3,4,5,6,7,8	4547	1.8389	14.3	81	9	10	15
11	ALIGN_000788	2,3,4,5,6,7,12,14	1629	0.7085	24.3	945	80	225	135
12	ALIGN_000832	2,3,4,5,6,7,9,10	1185	0.0000	10.0	327	50	49	315
13	ALIGN_000853	1,2,3,4,5,6,7,8	5307	3.3574	12.9	225	20	225	45
14	ALIGN_000930	2,3,4,5,6,7,8,9	1321	0.0000	78.6	10395	8925	10395	10393
15	ALIGN_000931	2,3,5,14,15,16,19,21	1231	0.8196	100.0	10395	9876	10395	10395
16	ALIGN_000984	2,3,4,6,7,11,12,13	1139	2.0933	45.7	10395	2344	10395	10391

The sequences taken from each alignment are listed in the second column. ΔAIC values for the Felsenstein84 nucleotide substitution model were calculated using PAUP* and ModelTest. The percentage of four-taxon subset for which the star topology was the ML solution (unresolved quartets) was calculated using TreePuzzle. The last four columns show number of trees out of possible 10395 included in the 0.95 confidence set using: expected likelihood weights (ELW) test, Shimodaira-Hasegawa (SH) test, generalised least squares (GLS) test, and weighted least squares test (WLS).

In general, we have observed that various methods gave expected results (confidence sets larger for WLS than for GLS, and closer to SH set size) when there was a large number of site patterns in the alignment (data sets 1, 6, 9 and 12), even when the percent of unresolved quartets was quite high (data set 1). However, it is not clear why for some data sets WLS gave a much more smaller confidence set than GLS, as can be observed for the data set 13, which is quite similar to 12 (for instance, the number of site patterns was 45 and 41, respectively) or data set 2, which is similar to 9 (both had 69 patterns). When the number of site patterns was very low (14–20) and the number of unresolved quartets extremely high (14, 15, 16), all tests gave similar results. It appears that larger confidence set for GLS than for WLS were observed especially when the number of patterns was low (around 30) but the high percentage of resolved quartets indicated good phylogenetic signal (data sets 5, 7 and 8). On the other hand, both LS methods gave similar results when the phylogenetic signal was worse (3, 4, 10), with the exception of data set 11.

The analysis of eight-species data sets discussed above shows the limitations of the GLS approach when the covariance matrix is close to singular. Indeed, both in simulations (not shown) and for biological sequences obtained from the EMBL-ALIGN database we observed that in many cases the calculation of the GLS statistic was not possible due to the singularity of the distance matrix. This was especially pronounced when the sequences were relatively short.

### The coverage of LS tree testing methods in simulations

Recent results of Shi et al. [26] indicated a strange behavior of the GLS test when the number of taxa increased in simulations. In this previous study [26], sequences were simulated over three ML topologies constructed from a 10-, 15- and 20-taxon subsets of a published 66-taxon tree of placental mammals [27]. Shi et al. observed that for the high number of taxa, although the number of trees included in the GLS confidence set increased, the method undercovered. The observed coverage of the 20-taxon tree was only 0.84 for 95% confidence set [26], lower than the lower bound for the nominal coverage, that is, the frequency in which the confidence set includes the true tree (this is approximately 0.91–1 for 0.95 nominal coverage; the lower bound can be estimated as c−1.645c(1−c)/100
 MathType@MTEF@5@5@+=feaafiart1ev1aaatCvAUfKttLearuWrP9MDH5MBPbIqV92AaeXatLxBI9gBaebbnrfifHhDYfgasaacH8akY=wiFfYdH8Gipec8Eeeu0xXdbba9frFj0=OqFfea0dXdd9vqai=hGuQ8kuc9pgc9s8qqaq=dirpe0xb9q8qiLsFr0=vr0=vr0dc8meaabaqaciaacaGaaeqabaqabeGadaaakeaacqWGJbWycqGHsislcqaIXaqmcqGGUaGlcqaI2aGncqaI0aancqaI1aqndaGcaaqaaiabdogaJjabcIcaOiabigdaXiabgkHiTiabdogaJjabcMcaPiabc+caViabigdaXiabicdaWiabicdaWaWcbeaaaaa@3D9E@ for a given nominal coverage *c *[26]).

**Figure 1:**
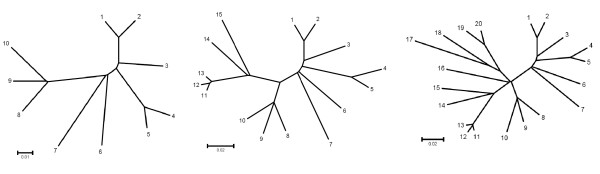
The 10-, 15- and 20-taxon trees used in the simulations.

In an effort to reproduce this simulation study, we have used the same tree topologies (presented in Fig. 1), and the same parameters for HKY substitution model (transition/transversion ratio 2.93, base frequencies A:0.37, C:0.24, G:0.12, T:0.27) to simulate 3000-nt sequences with EVOLVER (part of the PAML package [28]). To get the measure of the size of confidence set, for each simulated data set we have tested 100 trees chosen from the trees with the highest likelihood found by a heuristic search with the nearest-neighbor interchange using PAUP* [29]. The largest difference in log likelihood among those trees was 506.4, 399.8, and 156.5 for 10-, 15- and 20-taxon trees, respectively. In other words, we have chosen 100 trees from a larger spectrum of best trees (which resulted in larger maximum differences in log likelihood) then just the best 100 trees found in the heuristic search as in the previous study [26] (the maximum difference in log likelihood for the 100 best trees was 84.5 for 10-taxon trees, 46.1 for 15-taxon, but only 9.9 for 20-taxon trees).

Table 3 shows that as the number of taxa increased, so did the size of the confidence sets, for both the GLS and the WLS test (and indeed the other tests we employed, SH and ELW). This is expected, especially for the distance-based methods, considering that the trees used in the simulation have partially a star topology (many interior branches close to 0; Fig. 1). The size of the WLS confidence set was consistently larger than for the GLS test, indicating that WLS is more conservative. As reported by Shi et al. [26], we have observed that GLS undercovers; this behavior, however, was not observed for WLS, which always included the true tree in the 0.95 confidence set (and also in the 0.80 confidence set, not shown).

**Table 3:** The confidence sets and their coverage for the simulated data

**Number of taxa**	**Average number of trees out of 100 in the test set in the 0.95 confidence set **				**The coverage of 0.95 confidence set**	
	**SH**	**ELW **	**GLS **	**WLS**	**GLS**	**WLS**

10	19.28	5.26	5.32	14.98	0.84	1.00
15	14.43	3.65	8.30	14.84	0.78	1.00
20	57.69	32.48	52.04	95.43	0.77	1.00

The table shows the average number of trees (for 100 simulations) in the 95% confidence sets of GLS, WLS, SH and ELW out of 100 trees constructed for simulated 10-, 15- and 20-taxon sequences and the coverage of this confidence sets for GLS and WLS (the frequency in which the confidence set included the true tree).

### Large data set of Hepatitis C Virus sequences

The next data set we considered consisted of 295 short (532 nt) sequences obtained from 31 patients involved in the analysis of a Hepatitis C Virus (HCV) outbreak. This data set could not be analyzed with the existing implementations of the GLS procedure, even using the GLSDNA_EIG routine.

The sequences correspond to the E1–E2 region of the viral genome, which includes the hypervariable region 1 and codes for surface proteins. Out of 295 sequences under analysis, 277 were derived from 23 patients presumably involved in the outbreak, eight were from local controls unrelated to the outbreak, and ten sequences were obtained from external controls from different geographical origins. The TVM+Γ+I model (a restriction of the GTR model in which the two transition rates are equal) was used to calculate ML distances and branch lengths using PAUP* [29] and TREE-PUZZLE [25]. This model was found to be optimal according to the Akaike Information Criterion [30] using Modeltest 3.6 [24].

The test set consisted of 32 trees: the maximum likelihood tree and 31 trees in which the clone sequences of each single local patient in the analysis were forced to form a monophyletic clade with geographically unrelated sequences not belonging to the outbreak. Following an analogous reasoning to [31] and [32], if such tree is included in the confidence set, then the patient can be excluded from the outbreak, and vice versa: if this tree is excluded, then the patient can be assigned to the outbreak. Table 4 shows which trees were included in the confidence sets using the WLS method and the ELW, KH, and SH tests.

**Table 4:** Topology testing with a large data set of closely related Hepatitis C Virus sequences.

**patient tested**	**ELW**	**KH**	**SH**	**WLS**
none (ML tree)	**0.5646**	**1.0000**	**1.0000**	**1 **(24612.96)
LC-51	**0.0304**	**0.0830**	**0.9690**	**1 **(25425.09)
LC-86	**0.0232**	0.0430	**0.6840**	**1 **(25420.40)
LC-26	0.0057	0.0180	**0.4890**	**1 **(24920.98)
LC-24	0.0026	0.0090	**0.5670**	**1 **(24685.62)
LC-59	0.0051	0.0230	**0.6430**	**1 **(25201.22)
LC-53	**0.0823**	**0.1060**	**0.9790**	**1 **(24663.34)
LC-38	**0.1297**	**0.1640**	**0.7740**	**1 **(24183.58)
LC-63	0.0131	0.0210	**0.5010**	**1 **(25707.20)
EO-79	**0.0774**	**0.0750**	**0.8960**	**1 **(24590.74)
EO-47	**0.0313**	**0.0840**	**0.9700**	**1 **(25296.47)
EO-95	0.0009	0.0060	**0.3930**	**1 **(26175.50)
EO-12	0.0055	0.0260	**0.6230**	**1 **(23654.40)
EO-02	**0.0226**	**0.0620**	**0.7740**	**1 **(25392.13)
O1–00	0.0000	0.0000	0.0000	**1 **(39958.46)
O1–69	0.0057	0.0170	**0.4890**	**1 **(24920.98)
O2–60	0.0000	0.0000	0.0000	0 (56449.21)
O2–61	0.0000	0.0000	0.0000	**1 **(37982.68)
O2–29	0.0000	0.0000	0.0000	0 (64100.40)
O3–10	0.0000	0.0000	0.0000	0 (50280.91)
O3–72	0.0000	0.0000	0.0000	0 (57742.44)
O3–62	0.0000	0.0000	0.0010	0 (56689.96)
O3–63	0.0000	0.0000	0.0000	0 (58317.22)
O3–91	0.0000	0.0000	0.0000	0 (54015.96)
O3–12	0.0000	0.0000	0.0000	0 (63679.38)
O3–03	0.0000	0.0000	0.0000	0 (63107.46)
O3–42	0.0000	0.0000	0.0000	0 (62828.41)
O3–50	0.0000	0.0000	0.0000	0 (55717.93)
O3–54	0.0000	0.0000	0.0000	0 (45640.57)
O3–80	0.0000	0.0000	0.0010	0 (63024.57)
O3–79	0.0000	0.0000	0.0000	0 (58853.93)
O3–98	0.0000	0.0000	0.0000	0 (51672.80)

The data set consisted of 295 sequences corresponding to the E1–E2 region of the viral genome taken from 23 patients plus 8 local control (LC) sequences of the same genotype (HCV-1b) taken from individuals unrelated to the outbreak. The results from a more detailed analysis with an expanded data set were used to separate the 23 patients into four groups: EO, excluded from the outbreak; O1, involved in the outbreak, transmission chain 1; O2, involved in transmission chain 2; and O3, involved in transmission chain 3. The test set consisted of 32 trees, the ML tree and 31 trees in which the sequences from each patient where moved to form a monophyletic group with the external controls. For each alternative topology the probability associated to the corresponding test statistic (see abbreviations in Table 1) is shown. Topologies included in the confidence set around the ML tree at the 0.05 level (bold) are indicated. For WLS, the value of the corresponding statistic is shown between parentheses.

As a reference we have used the results obtained in a more detailed analysis (Bracho et al., in preparation) of an expanded data set derived from the same patients, which included clone sequences from the same E1–E2 region and direct sequences from the Ns5b genomic region. This analysis indicated that 18 of the 23 patients belonged to the outbreak and allowed to identify three independent transmission events, one involving 13 patients, and two smaller transmission chains, with 3 and 2 patients, respectively. Using these results as a standard, all tests correctly identified the isolates belonging to the 13-patient transmission chain (they correspond to the last 13 entries in Table 4, denoted by prefix O3-), because in all cases the modified tree was excluded from the confidence set of trees. Out of the 5 additional isolates also belonging to the outbreak according to the reference analysis (identified by prefixes O1- and O2- in Table 4), two (O2–60 and O2–29) were identified by all tests as belonging to the outbreak, one (O1–69) was excluded from the outbreak by SH and WLS tests, while the remaining two patient isolates (O1–00 and O2–61) were excluded from the outbreak only by the WLS test. On the other hand, the ELW and KH tests both assigned to the outbreak several local control patients and also patients which were judged external by the reference analysis (EO-12 and EO-95). This suggests that although the ELW and KH are more powerful, their results are less reliable since they erroneously include in the outbreak patients which do not belong to it according to other evidence.

**Figure 2:**
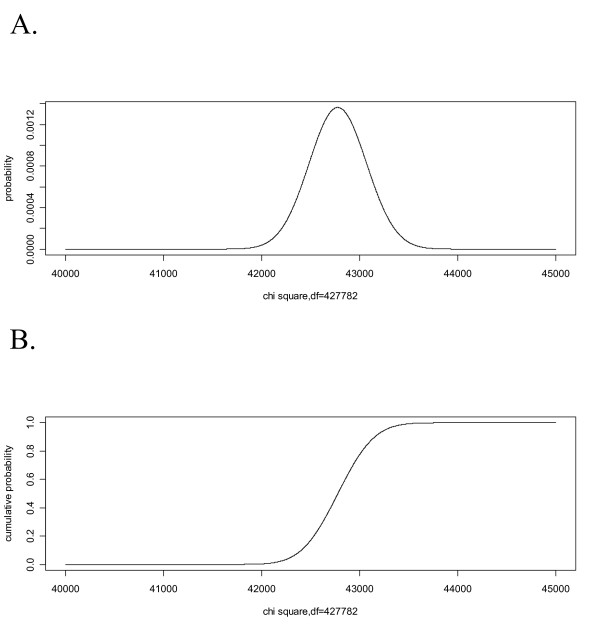
**Shape of the chi square distribution with 42778 degrees of freedom**. Panel A shows the density distribution; panel B the cumulative density.

We note that WLS shows *P*-values close either to zero or one, which at first may be striking. The explanation is quite simple. The number of branch lengths grows linearly with the number of species in a bifurcating tree, and the number of distances is quadratic with the number of species, and so is the number of degrees of freedom of the chi-square distribution. The consequences can be observed in Fig. 2, which shows the shape of the chi-square distribution for 42778 degrees of freedom and makes clear that the values very close to zero or one would be observed for any LS test procedure.

### DNA/DNA hybridization data

The advantage of LS methods for tree reconstruction is not only their high computational efficiency but also that the data need not to be characters: distances that are not derived directly from sequences can also be used for tree reconstruction. Also, occasionally access to the original character data derived from sequences is not possible; only distance methods can be used in such conditions. The WLS method of topology testing could also be used for such data provided that the variances are known or can be estimated. One example of such data is DNA/DNA hybridization data.

**Figure 3:**
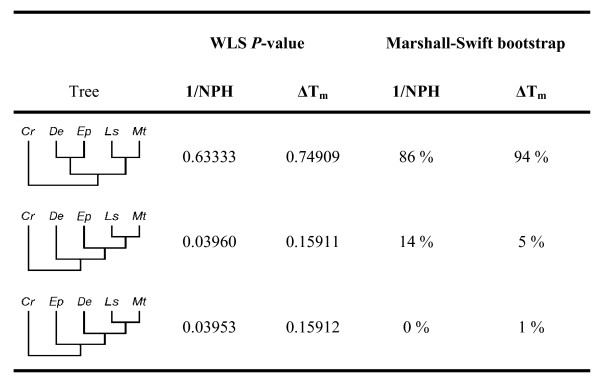
The analysis of sea dollar DNA/DNA hybridization data using the WLS method compared with the results of bootstrap [33].

The data set we will use here as an example was originally obtained by Marshall and Swift [33] using four species of sand dollars: *Dendraster excentricus *(Eschscholtz) (*De*), *Echinarachnius parma *(Lamarck) (*Ep*), *Leodia sexiesperforata *(Leske) (*Ls*), and *Mellita *spp. (*Mt*), with sea biscuit *Clypeaster rosaceus *(Linne) (*Cr*) as outgroup. This is a high quality data set of DNA/DNA hybridization data; although the normalized percent hybridization (NPH) values were all below 50%, the values were highly reproducible [33], and the data reported in the original paper included the variances for two distances measures: 1/NPH (the averaged inverses of normalized percent hybridization), and ΔT_m _(the averaged differences in melting temperatures), corrected for multiple substitutions using the Jukes-Cantor [34] formula. All 15 possible five-species trees were considered using the WLS test; Fig. 3 shows the results, compared with the bootstrap [16]. Both methods rejected 12 topologies that were not shown in the figure. For the 1/NPH distances, only one tree belongs to the WLS 0.95 confidence set, the bootstrap confidence set included one additional topology. Both methods gave similar results also for the ΔT_m _data: three (WLS) or two (bootstrap) topologies. The quality of the regression used to calculate the two parameters necessary for the calculation of the WLS statistic was not as good as is routinely observed for sequence data (where regression coefficients are often close to 0.99): R^2 ^was 0.79 for the 1/NPH data, but only 0.22 for the ΔT_m _data. However, when the whole variance matrix was used in the calculations (instead of using only regression parameters to calculate the WLS statistic), the sizes of WLS 0.95 confidence sets did not change (not shown).

## Discussion

Distance methods for tree inference have the advantage that they can be used when the distances are not derived from characters or when there is no access to the original sequence data. When the data are sequences, using the distance matrix unavoidably results in some loss of information. This leads to reduced statistical power compared to character-based methods such as maximum likelihood or Bayesian methods of tree reconstruction, the advantage being a much higher computational efficiency, an important issue for very large data sets. Contrary to maximum parsimony, distance methods have been shown to be consistent, and they usually are as accurate as maximum likelihood methods [35]. In particular, the least squares (LS) criterion is well-established both for phylogenetic reconstruction [12,15,36-38] and hypothesis testing [12,13]. Simulation studies [35,38] have shown that the WLS method is accurate and consistent for tree inference.

Both GLS and WLS are based on the assumption that distances are normally distributed. Although it has been traditionally considered that this assumption is not fulfilled for distances derived from nucleotide or amino acid sequences [39], more recent work [13] showed that provided the distances are maximum likelihood estimates, their distribution is approximately multivariate normal, which led to the proposal of a GLS test for topologies. The LS method is applicable to distance measures not necessarily derived from sequence data, but whenever it is reasonable to assume normality of the distances. What makes the WLS test different from the GLS is that distances are considered independent for computational reasons. Although the phylogenetic distances are not independent because taxa share common evolutionary history, our previous results suggest that this does not affect the performance of the WLS method for branch testing [6].

In this work we have investigated the applicability of LS methods for topology testing and the consequences of using the WLS approximation. To compare our method to previously proposed procedures, we first restrict the discussion to examples in which the distances were derived from sequences and the variances estimated by bootstrapping. However, the WLS method is applicable for testing topological hypotheses and for finding confidence sets of trees as long as the distance matrices and at least some of the variances associated with the distances are known. The distances may, for example, be derived from DNA-DNA hybridization assays, or result from averaging different data sets.

The first data set we considered, that of mammalian mitochondrial proteins, has gained a *de facto *benchmarking status, and has been discussed in a number of previous studies [3,8,10,11,13]. In these studies, restricting the number of topologies supported by the data was at least implicitly considered desirable. Taking into account that various methods give different answers and that the topology believed to be the best estimate [40] is not included in the 0.95 confidence set by the ELW and KH tests, it might be argued that this is not necessarily so. This topology was not included neither in the confidence sets obtained with SOWH nor the Approximate Unbiased test [3,8,10,11,13]. However, erroneous rejections in the analyses of real data may be caused by bias, not only because the confidence sets are too radical. Although conservative confidence sets may be useful in face of unrecognized biases (the models of molecular evolution are inevitably too simple), it is hard to investigate the effects of biased estimates on the performance of various methods.

One way around this problem is to investigate the performance of the methods using simulated data. In the case of our simulations, the number of possible sources of bias was diminished (for example, the substitution model used to simulate the data was used also when testing, and no heterogeneity among the sites was introduced). The disadvantage is, of course, that the method that performs best in simulations may not necessarily work well in real data analysis. Therefore, we first investigated the size of confidence sets for topologies obtained with real data: eight-taxon nucleotide data sets from the EMBL-ALIGN database. The results were somewhat surprising. The WLS confidence sets were always smaller than the SH sets and, what is more surprising, in the majority of cases smaller than the confidence sets obtained using the GLS method. Perhaps numerical errors or errors in the GLS implementation may account for these rather unexpected outcomes, although it appears that such discrepancies may occur principally when the number of site patterns in the sequence alignment is low. For simulated data, strange behavior of the GLS method has been previously reported [26]: the true tree was included in the confidence set rarer than expected, especially as the number of taxa in the trees increased. This behavior was observed even though the number of trees included in the confidence set grew larger with the increase of the number of taxa for the topology used in the simulations (close to the star tree). In the same simulations, WLS always included the true tree in the confidence set, which is an expected conservative behavior considering on the one hand the construction of the test and on the other the topology of the trees.

When the number of taxa is very large, the fact that *P*-values are being indistinguishable either from zero or from one may at first look striking. This problem is not a result of the computational differences between WLS and GLS, but rather the shape of the chi-square density distribution (Fig. 2). Therefore, the GLS method would also result in extreme *P*-values, if it could be applied to the HCV data set, for which the covariance matrix was very close to singular due to the presence of very closely related sequences.

The covariance matrix may be non-invertible for two reasons: the variance of one or more distances is practically zero or the correlations between the distances are (almost) perfect. Both conditions are related and occur when the data set includes very closely related taxa: small distances would have very small variances, and the distances between these taxa and the other OTUs would be highly correlated (and thus far from being independent). In fact, both conditions also affect the WLS statistic; in the first case, this is caused by division by values very close to zero (the computation of the WLS statistic involves division by variance). However, the results obtained with WLS for the HCV data set were reasonable. This might be due to an over-estimation of small variances. Since in theory the true variances should be used, the WLS statistic would be smaller (after division by a larger value) and the test more conservative. Indeed, in the HCV-1b E1–E2 example the WLS test gave more conservative estimation of the tree confidence set than the SH test. However, the comparison with the results obtained from an independent analysis indicates that these results were reliable.

A result which might strike as paradoxical is the rejection of all the possible topologies by LS methods, rarely observed for short sequences, but unavoidable for very long sequences, both real and simulated (our unpublished observations). This may occur because a particular data set indeed cannot be fitted to a tree (and would be better represented by a network). However, as the number of characters increases, the variance decreases, leading to the rejection of all the hypotheses. Indeed, it is well known that *P*-values are dependent on sample size [41], and that one can always reject a null hypothesis with a large enough sample, even if the true difference is trivial (the so called Lindley's [42] paradox).

## Conclusion

In this work, we have explored the limitations of LS methods for phylogeny testing. The advantage of these methods is their high computational efficiency, which allows their application to very large data sets. We have proposed a way to approximate the value of the test statistic (the WLS method) which requires only a matrix of distances and at least some of the variances. In principle, this allows the application of the method for data sets in which the distances are not derived directly from sequences (or for which the sequence data is not available). We have shown the applicability of the method to such data (DNA-DNA hybridization data set), but only by considering sequence data we have been able to compare the results of the WLS method to other methods for construction of tree confidence sets, including the GLS method. We believe that none of these methods is free from limitations, and the fact that in practical applications various tests give contradictory results has been noticed previously [10,11]. This results in an uncomfortable situation because it is not very difficult to simulate data in such a way that they show the superiority of a particular test over others and, in which by choosing either a 'more conservative' or 'less conservative' test, one can accept or reject a particular topological hypothesis.

The results obtained with the WLS method we present are reasonable in the sense that they are similar to the results obtained with other tests. This cannot be said of the available implementation of the GLS method: firstly, for many real data sets the test statistic cannot be calculated. It is difficult to say if the second problem (all the trees in the confidence set in spite of a strong phylogenetic signal) is or not caused by errors in the implementation. The WLS method we propose is computationally very efficient and is not restricted to a particular substitution model. It may be useful to assess the phylogenetic signal in the data, and to screen out the hypotheses which are likely to be rejected by more powerful tests or when few alternatives are available (as in the DNA-DNA hybridization example).

## Methods

It has been shown [13] that provided the distances derived from sequences satisfy the maximum likelihood criterion, their distribution is approximately multivariate normal, which allows to estimate their variances and covariances using the sample average method [13]. Under the null hypothesis that a given topology is true, the GLS statistic (eq. 1) follows the chi-square distribution [13]. The number of degrees of freedom corresponds to the number of entries in the distance matrix minus the number of branches estimated in the tree. If the tree is fully bifurcating and incorporates T entries, this corresponds to T(T - 1)/2 - (2T - 3). Calculating the GLS statistic and then the corresponding *P*-values from the chi-square distribution allows to sort a set of competing topologies and to establish confidence sets for topologies. In other words, as long as the estimates of the entries in the covariance matrix are consistent, provided that the number of sites is large, over the long run the *P*-values corresponding to true topologies will be larger than the significance threshold *α*, and the true topologies will be included in the confidence sets in a fraction (1-*α*) of the analyses.

However, this reasoning assumes that both the distances and the variances are well-estimated. Even if the estimation method is consistent, in practice the number of sites may not be large enough for the estimates to be precise. Using an inappropriate substitution model may lead to bias. Finally, estimating a large numbers of parameters (covariances) from limited data may lead to large errors in the estimates.

Additionally, some data sets present particular computational problems. When the distance matrix is large and contains closely related taxa, the covariance matrix may be close to singularity [6]. Ignoring very small entries in the matrix leads to a more conservative test (the test statistic is smaller, which results in higher *P*-values). In the extreme, when all the covariances are ignored, the computational problems associated with inverting the covariance matrix are avoided, which leads to the WLS statistic. We have previously presented a further simplification of the WLS approach in which only two parameters were used instead of T(T-1)/2 variances of distances between T taxa [6]. Briefly, the variances can be approximated by σ(p)−2dij−p
 MathType@MTEF@5@5@+=feaafiart1ev1aaatCvAUfKttLearuWrP9MDH5MBPbIqV92AaeXatLxBI9gBaebbnrfifHhDYfgasaacH8akY=wiFfYdH8Gipec8Eeeu0xXdbba9frFj0=OqFfea0dXdd9vqai=hGuQ8kuc9pgc9s8qqaq=dirpe0xb9q8qiLsFr0=vr0=vr0dc8meaabaqaciaacaGaaeqabaqabeGadaaakeaaiiGacqWFdpWCdaqhaaWcbaGaeiikaGIaemiCaaNaeiykaKcabaGaeyOeI0IaeGOmaidaaOGaemizaq2aa0baaSqaaiabdMgaPjabdQgaQbqaaiabgkHiTiabdchaWbaaaaa@3A33@, where the parameters σ(p)2
 MathType@MTEF@5@5@+=feaafiart1ev1aaatCvAUfKttLearuWrP9MDH5MBPbIqV92AaeXatLxBI9gBaebbnrfifHhDYfgasaacH8akY=wiFfYdH8Gipec8Eeeu0xXdbba9frFj0=OqFfea0dXdd9vqai=hGuQ8kuc9pgc9s8qqaq=dirpe0xb9q8qiLsFr0=vr0=vr0dc8meaabaqaciaacaGaaeqabaqabeGadaaakeaaiiGacqWFdpWCdaqhaaWcbaGaeiikaGIaemiCaaNaeiykaKcabaGaeGOmaidaaaaa@32B0@ and *p *(power of the sum of squares) correspond to the slope and ordinate at the origin of the linear regression

ln *σ*^2^_*ij *_= ln *σ*^2^_(*p*) _+ *p *ln *d*_*ij*_

of the logarithm of distance variances (σij2
 MathType@MTEF@5@5@+=feaafiart1ev1aaatCvAUfKttLearuWrP9MDH5MBPbIqV92AaeXatLxBI9gBaebbnrfifHhDYfgasaacH8akY=wiFfYdH8Gipec8Eeeu0xXdbba9frFj0=OqFfea0dXdd9vqai=hGuQ8kuc9pgc9s8qqaq=dirpe0xb9q8qiLsFr0=vr0=vr0dc8meaabaqaciaacaGaaeqabaqabeGadaaakeaaiiGacqWFdpWCdaqhaaWcbaGaemyAaKMaemOAaOgabaGaeGOmaidaaaaa@324D@) on the logarithm of observed distances. The distance variances can be part of the original data (for example, when DNA hybridization data are considered). They can also be estimated by bootstrapping the character matrix.

### Implementation

The program WeightLESS [6], originally written to allow for interior branch testing using the WLS likelihood ratio test, has been modified to calculate also the *P*-values corresponding to each topology in the tree input file. The distances between taxa are input in a separate file. The user may provide the two parameters necessary for the calculation of the WLS statistic or they can be estimated by the program if many distance matrices are provided (again, in a separate file). For sequence data, this file may be constructed by calculating pseudo distance matrices using bootstrapping. Alternatively, the whole variance matrix can be used in the calculations. The program (the C source code, the documentation, and binaries for Linux and DOS/Windows) is available at the author's webpage [43].

## Authors' contributions

AC carried some of the computational analysis. RS was involved in the development of the method, while FG was involved in the analysis of the viral data. Both critically revised the manuscript. BW designed the study, implemented the method in C and prepared the documentation of the program, carried part of the analysis, and drafted the manuscript. All authors read and approved the final manuscript.
